# Diagnostic value of loop‐mediated isothermal amplification assay for hand, foot, and mouth disease

**DOI:** 10.1002/jcla.23776

**Published:** 2021-04-01

**Authors:** Ying‐Zhou Chen, Zhi‐Qing Zhan, Li‐Quan Zhou, Min‐Shan Chen, Xun‐Jie Cao, Ya‐Ping Li, Xu‐Guang Guo

**Affiliations:** ^1^ Department of Clinical Laboratory Medicine The Third Affiliated Hospital of Guangzhou Medical University Guangzhou China; ^2^ Department of Clinical Medicine The First Clinical School of Guangzhou Medical University Guangzhou China; ^3^ Department of Clinical Medicine The Third Clinical School of Guangzhou Medical University Guangzhou China; ^4^ Department of Clinical Medicine The Second Clinical School of Guangzhou Medical University Guangzhou China; ^5^ Key Laboratory for Major Obstetric Diseases of Guangdong Province The Third Affiliated Hospital of Guangzhou Medical University Guangzhou China; ^6^ Key Laboratory of Reproduction and Genetics of Guangdong Higher Education Institutes The Third Affiliated Hospital of Guangzhou Medical University Guangzhou China

**Keywords:** diagnosis, hand foot and mouth disease, loop‐mediated isothermal amplification, meta‐analysis, polymerase chain reaction

## Abstract

**Background:**

Nowadays, hand, foot, and mouth disease (HFMD) has a significant negative impact on children's health, especially in the Asia‐Pacific region. Loop‐mediated isothermal amplification assay (LAMP) is a highly efficient and convenient novel tool. However, its diagnostic accuracy for HFMD is still not clear. Therefore, we conducted a meta‐analysis in order to evaluate the potential of LAMP assay for the diagnosis of HFMD, in which the reference standard was polymerase chain reaction (PCR).

**Methods:**

A protocol was predetermined (CRD42020212882) in PROSPERO. We retrieved seven databases including PubMed for relevant studies published before October 2020. Articles were included if they compared the diagnostic efficiency of LAMP with PCR for HFMD through detecting clinical samples which was more than 15. Statistical analysis was performed by STATA 15.1 software. Risk of bias and applicability were assessed using Quality Assessment of Diagnostic Accuracy Studies. No funding was used for the study.

**Results:**

A total of 18 retrospective studies including 2495 samples from China were finally included. Reference standards of them included RT‐PCR and non‐RT‐PCR. The merged sensitivity and specificity with 95% confidence interval (95% CI) were 1.00 (0.97–1.00) and 0.97 (0.88–0.99), respectively. The pooled PLR, NLR, and DOR with 95% CI were 11.17 (5.91–21.11), 0.05 (0.03–0.09), and 538.12 (183.17–1580.83), respectively. The AUC of SROC was 1.00 (95% CI: 0.99–1.00).

**Conclusion:**

In conclusion, our research revealed high sensitivity and specificity of LAMP in diagnosing HFMD. However, more high‐quality research is required to prove this conclusion.

## Graphical abstract

Our research revealed high sensitivity and specificity of LAMP in diagnosing HFMD. The AUC of SROC was 1.00 (0.99–1.00), indicating the high diagnostic accuracy of LAMP for HFMD.

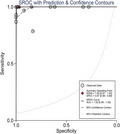

## 1 INTRODUCTION

Hand, foot, and mouth disease (HFMD) is a common infectious disease in children caused by Enterovirus A, in which the most generally reported genotypes are Enterovirus A71 (EV‐A71) and Coxsackievirus A6, A10, and A16 (CVA6, CV‐A10, and CV‐A16).^1^ It is common in preschoolers. In China, children below the age of 3 are most susceptible to HFMD.^2^ HFMD is characterized by macular papules and herpes on the palms, soles, oral mucosa, and buttocks, with or without fever.^3^ It is prone to central nervous system damage and severe cases of brainstem encephalitis, leading to neurogenic pulmonary edema, pulmonary hemorrhage, and even death.^4^ Timely and correct diagnosis is of great significance for its treatment.

The traditional laboratory diagnostic methods of HFMD are mainly isolation culture and serological detection. However, because of the need for a particular culture medium, time‐consuming and complex experimental conditions, and comparatively low detection rate leading to late diagnosis, isolation culture has not been routinely used in HFMD diagnosis. The practical value of serology in the diagnosis of early HFMD infection is limited since serological diagnosis is time‐consuming.^5^ It is usually used as a retrospective diagnosis and epidemiological investigation to provide the basis for the formulation of prevention and control measures.^6^ In recent years, with the rapid development of molecular biotechnology, real‐time fluorescent quantitative polymerase chain reaction (PCR) has become a routine method for laboratory diagnosis of HFMD.^7^, ^8^ However, a lot of money is required in the early stages to meet the needs of the environment, personnel, and instruments, so it is difficult to detect this in a primary laboratory. With the shortcoming of requiring costly precise equipment, PCR may not be a good method to diagnose HFMD in the basic clinical place, especially for developing countries.^9^ Therefore, it is necessary to find a rapid, economical, simple, sensitive, and specific tool to diagnose HFMD.

Loop‐mediated isothermal amplification (LAMP) is a newly emerged nucleic acid amplification method, which relies upon DNA polymerase with strand replacement ability and two pairs of primers, which can identify six regions of the target sequence.^10^ The whole detection reaction can be completed only at 65℃ for 1–2 h, and the results can be judged by the naked eye. Compared with other detection methods, this method is simple to operate and the equipment used is not expensive.^11^ The most prominent feature of LAMP is that it allows instant diagnosis.^12^ It has a widespread application in the diagnosis of infectious diseases such as malaria and trypanosomiasis.^13^ Moreover, it has been regarded as a potential alternative to PCR in the laboratory.^14^ However, the diagnostic accuracy of LAMP for HFMD diagnosis remains unclear. Hence, we performed a pooled‐analysis to evaluate the overall efficacy of LAMP in diagnosing HFMD. PCR was served as the reference standard.

## 2 MATERIALS AND METHODS

### 2.1 Protocol and registration

A protocol was predetermined in PROSPERO, and the registration number was CRD42020212882, which could be accessed at https://www.crd.york.ac.uk/prospero/display_record.php?ID=CRD42020212882. This research was conducted following Preferred Reporting Items for a Systematic Review and Meta‐analysis of Diagnostic Test Accuracy Studies (PRISMA‐DTA).^15^

### 2.2 Search strategy

We identified all the studies published before October 2020 by systematically searching Embase, PubMed, Cochrane Library, Web of Science, Wan Fang Data, SinoMed, and the Chinese National Knowledge Infrastructure (CNKI) databases using the following strategy as follows: (“Hand, Foot, Mouth Disease” OR “Hand, Foot and Mouth Disease” OR “hand foot and mouth disease” OR “hand foot mouth disease”) AND (“LAMP” OR “isothermal amplification loop‐mediated” OR “loop‐mediated isothermal amplification” OR “loop mediated isothermal amplification”). Then, we inspected the bibliographies of all the publications to complement the retrieval.

### 2.3 Study selection

Two review authors carefully reviewed all the retrieved articles and selected the articles according to the inclusion and exclusion criteria established in advance. If they had different opinions on some articles, they discussed them with a third review author until they were in agreement.

### 2.4 Inclusion and exclusion criteria

Articles were included in the analysis if (1) clinical samples of human were analyzed; (2) they compared the diagnostic accuracy of LAMP with PCR for HFMD; (3) PCR was served as the reference standard; (4) the sample size was more than 15; (5) the generated data sufficed to construct two‐by‐two tables including true positive (TP), false positive (FP), false negative (FN), and true negative (TN) for working out the sensitivity, specificity, and likelihood ratios.

Criteria for excluding the studies were as follows: (1) Duplicate publications; (2) Abstracts, case reports, letters, reviews, and editorials.

### 2.5 Data extraction

The following information was extracted individually by two review authors from the articles selected for inclusion: Publication information, sample size, specimen type, gold standard, and virus detected type. Data for 2·2 tables (TP, FP, FN, and TN) were extracted. When inconsistencies were encountered, the two review authors resolved them by reaching an agreement with a third one.

### 2.6 Quality assessment

Relying on the Quality Assessment of Diagnostic Accuracy Studies (QUADAS‐2) guidelines,^16^ two review authors assessed the risk of bias from 4 aspects: Patient selection, indicator testing, gold‐standard method, and timing and flow. They also considered the applicability concerns from the first three aspects. The final figure was made by Review Manager 5.3.

### 2.7 Statistical analysis

Stata 15.1 software was employed to perform the statistical analysis. We calculated the proportion of heterogeneity likely due to threshold effect and drew the summary receiver operating characteristic (SROC) curve to tell whether there was a threshold effect. Next, we calculated the merged diagnostic odds ratio (DOR), sensitivity, specificity, positive likelihood ratio (PLR), and the negative likelihood ratio (NLR) of the included studies and corresponding 95% confidence intervals (95% CI). We used Cochran's *Q* test and *I* ^2^ test to evaluate the heterogeneity derived from the non‐threshold effect of the eligible studies. If the *p* value of *Q* test >0.05 or *I* ^2^ < 50%, a fixed‐effects model was applied. Otherwise, we adopted a random‐effect model. Meta‐regression analysis was also conducted to validate whether heterogeneity was caused by different types of samples, reference standards, or the kinds of virus detected. The area under the SROC curve (AUC) was calculated. We plotted Deeks’ funnel plot to detect publication bias.

### 2.8 Subgroup meta‐analysis

Subgroup analysis was carried out depending on whether the standard reference was reverse transcription polymerase chain reaction (RT‐PCR) and on the types of samples, respectively.

## 3 RESULTS

### 3.1 Literature searching result

A total of 422 studies were identified from which 281 duplicates were discarded. After screening their titles and abstracts, 119 articles were excluded, and 22 articles were left to browse the full‐text. Finally, three articles were removed because of the lack of gold standard or due to insufficient data to construct a 2×2 table. Another article was discarded since the index test was combined with additional techniques. The remaining 18 studies^9^, ^33^ which were eligible were included to conduct pooled‐analysis. The flow diagram is shown in Figure S1.

### 3.2 Characteristics of the eligible studies

All these eligible articles were retrospective studies with a total of 2495 samples from China. These 18 articles were published between 2011 and 2017. The sample size ranged from 20 to 678. Funding Sources of these articles did not include related reagent makers. Seven^9^, ^18^, ^20^, ^24^, ^27^, ^28^, ^32^ of them included clinical samples from suspected patients with HFMD while eleven of them included clinical samples from confirmed patients. All the patients had symptoms associated with HFMD. Five^17^, ^18^, ^20^, ^21^, ^25^ of them generated data from only fecal samples while three^29^, ^31^, ^32^ of them reported data from only pharyngeal swabs. The rest used a variety of samples. Besides, eight^9^, ^18^, ^19^, ^22^, ^24^, ^25^, ^27^, ^33^ of them detected only Human enterovirus 71 (EV71), and two^21^, ^23^ of them detected Coxsackievirus A16 (CA16). The remaining 8 examinations detected both EV71 and CA16. The baseline characteristics are summarized in Table 1.

**TABLE 1:** Characteristics of the included studies

First author	Year	country	study design	reference standard	Sample size	sample type	virus type	TP	FP	FN	TN
Zhao	2011	China	retrospective	PCR	60	Fecal samples	EV71&CA16	34	4	0	22
Xia	2011	China	retrospective	RT‐PCR	108	Fecal samples	EV71&CA16	87	14	0	7
Jiang	2011	China	retrospective	rRT‐PCR	40	Fecal samples	EV71	26	0	2	12
Zong	2011	China	retrospective	rRT‐PCR	33	Fecal samples	CA16	7	0	0	26
Geng	2011	China	retrospective	RT‐PCR	58	Fecal samples Pharyngeal swabs	EV71	20	13	0	25
Shi	2011	China	retrospective	PCR	122	Fecal samples Pharyngeal swabs Vesicular fluids	EV71	56	2	0	65
Nie1	2012	China	retrospective	qRT‐PCR	145	Nasopharyngeal swab	EV71	112	0	17	16
Yan	2012	China	retrospective	RT‐PCR	68	Fecal samples	EV71	50	13	0	5
He1	2012	China	retrospective	rRT‐PCR	33	Fecal samples Throat swards rectal swards vesicular fluid	EV71	24	0	0	9
He2	2012	China	retrospective	rRT‐PCR	33	Fecal samples Throat swards rectal swards vesicular fluid	CA16	7	0	0	26
Nie2	2013	China	retrospective	rPCR	515	Fecal samples Pharyngeal swabs Liver swab CSF	EV71&CA16	336	2	0	177
Wang	2014	China	retrospective	RT‐PCR	36	Fecal samples Pharyngeal swabs	EV71	23	5	0	8
Ding	2014	China	retrospective	qRT‐PCR	261	unclear	EV71&CA16	169	0	17	75
Dai	2014	China	retrospective	qRT‐PCR	93	Pharyngeal swabs	EV71&CA16	80	2	0	11
Guan	2015	China	retrospective	rRT‐PCR	92	unclear	CVA16&other enteroviruses	9	0	0	83
Sun	2015	China	retrospective	RT‐PCR	20	Pharyngeal swabs	EV71&CA16	11	1	3	5
Gao	2016	China	retrospective	rPCR	678	Pharyngeal swabs	EV71&CA16	251	1	0	427
Li	2017	China	retrospective	RT‐PCR	100	Fecal samples Pharyngeal swabs	EV71	60	3	2	35

Abbreviations: CA16, Coxsackievirus A16; CSF, cerebrospinal fluid; EV71, Human enterovirus 71; FN, false negative; FP, false positive; PCR, polymerase chain reaction; qRT‐PCR, real‐time quantitative reverse transcription polymerase chain reaction; rRT‐PCR, real‐time reverse transcription polymerase chain reaction; RT‐PCR, reverse transcription polymerase chain reaction; TN, true negative; TP, true positive.

### 3.3 Quality assessment

**FIGURE 1:**
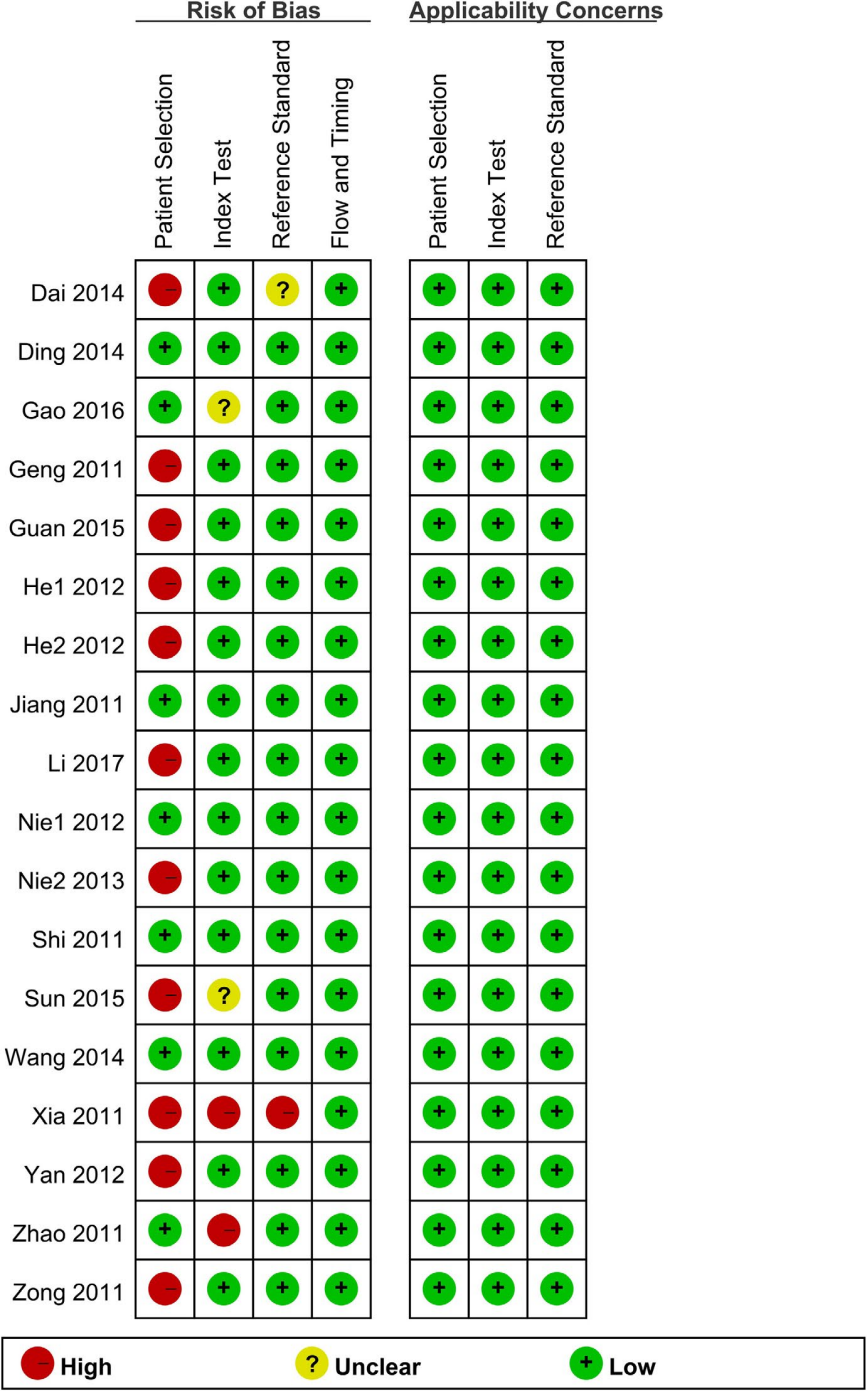
Quality assesstion graph of the included studies

**FIGURE 2:**
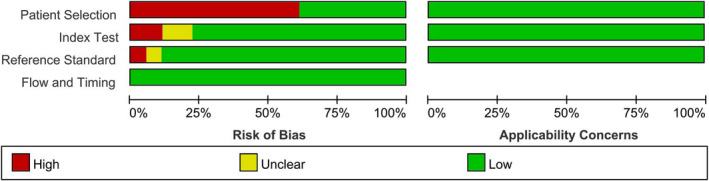
Quality assesstion summary of the included studies

Most studies had a low risk of bias in most domains. And they all had low concern for applicability. However, 11 (61.1%) of the studies were considered to have a high risk of bias in the patient selection domain due to the inclusion of participants with confirmed diagnoses. Risk of bias of another two studies^17^, ^20^ was assessed as high in the index test domain because they did not pre‐specify a threshold. Reference standard results of Xia's study^17^ were not interpreted without knowledge of the results of the index test. Therefore, this study was considered as high risk in the reference standard domain. The results of independent studies and the overall results are shown in Figures 1 and 2, respectively.

### 3.4 Threshold effect

**FIGURE 3:**
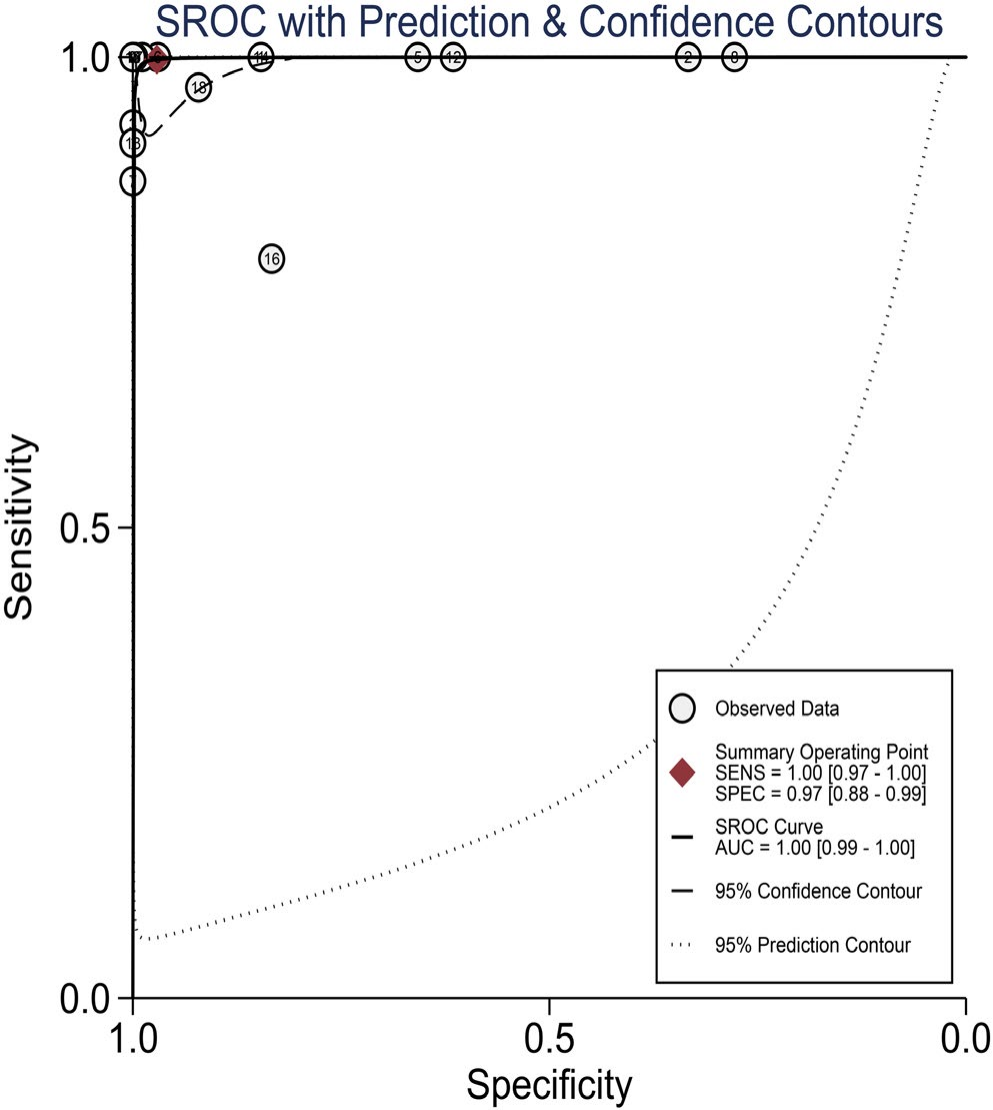
Summary receiver operating characteristic (SROC) curves of HFMD diagnosed by LAMP

“Shoulder‐arm” distribution of scatter plots did not exist in the SCOR curve (Figure 3). The proportion of heterogeneity likely due to threshold effect was 0.17. There was probably no threshold effect in the included studies.

### 3.5 Heterogeneity analysis of the non‐threshold effect

The non‐threshold effect was explored mainly using DOR. As shown in Figure S2, *I* ^2^ = 56.2% (*I* ^2^ > 50%) and *p* = 0.002 (*p* < 0.05), which suggested that the heterogeneity caused by non‐threshold effect existed. The random‐effect model was adopted to conduct the meta‐analysis. Nevertheless, high heterogeneity was also discovered through other indicators: sensitivity (*I* ^2^ = 94.0%, *p* < 0.05), specificity (*I* ^2^ = 95.9%, *p* < 0.05), PLR (*I* ^2^ = 88.9%, *p* < 0.05), and NLR (*I* ^2^ = 52.9%, *p* < 0.05).

### 3.6 Merge analysis results

To assess the diagnostic efficacy of LAMP assay using a random‐effect model, 18 sets of fourfold table data from 18 studies were merged. The pooled DOR was 538.12 (95% CI: 183.17–1580.83). The merged PLR, NLR, sensitivity, specificity, and corresponding 95%CI were 11.17 (5.91–21.11), 0.05 (0.03–0.09), 1.00 (0.97–1.00), and 0.97 (0.88–0.99). The results were presented in Figures S2–S4, and 4.

**FIGURE 4:**
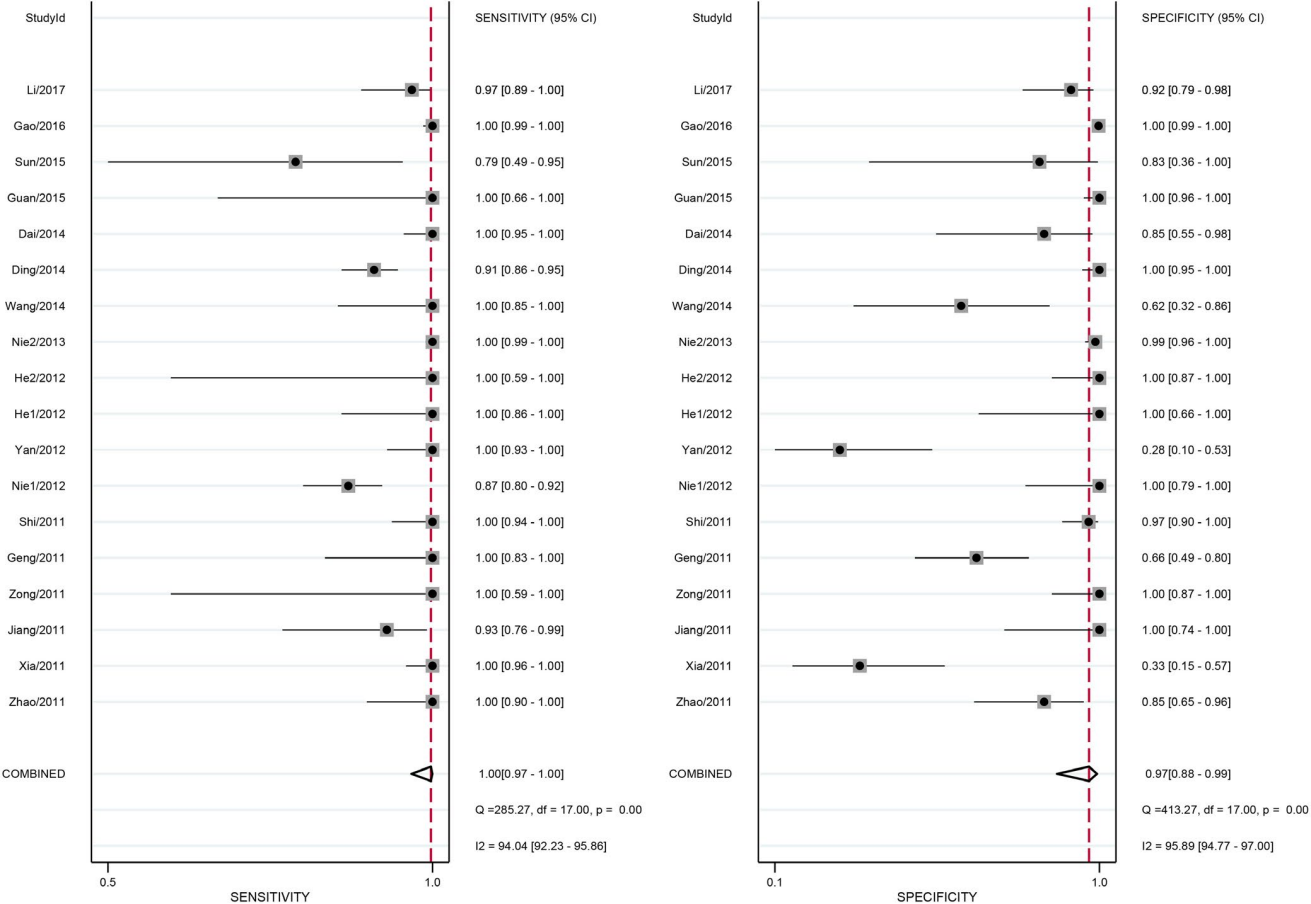
Forest plots for the pooled sensitivity and specificity of LAMP

### 3.7 SROC curve

The calculated AUC was 1.00 (95% CI: 0.99–1.00) as presented in Figure 3, indicating the high diagnostic accuracy of LAMP for HFMD.

### 3.8 Meta‐regression analysis and subgroup analysis

**FIGURE 5:**
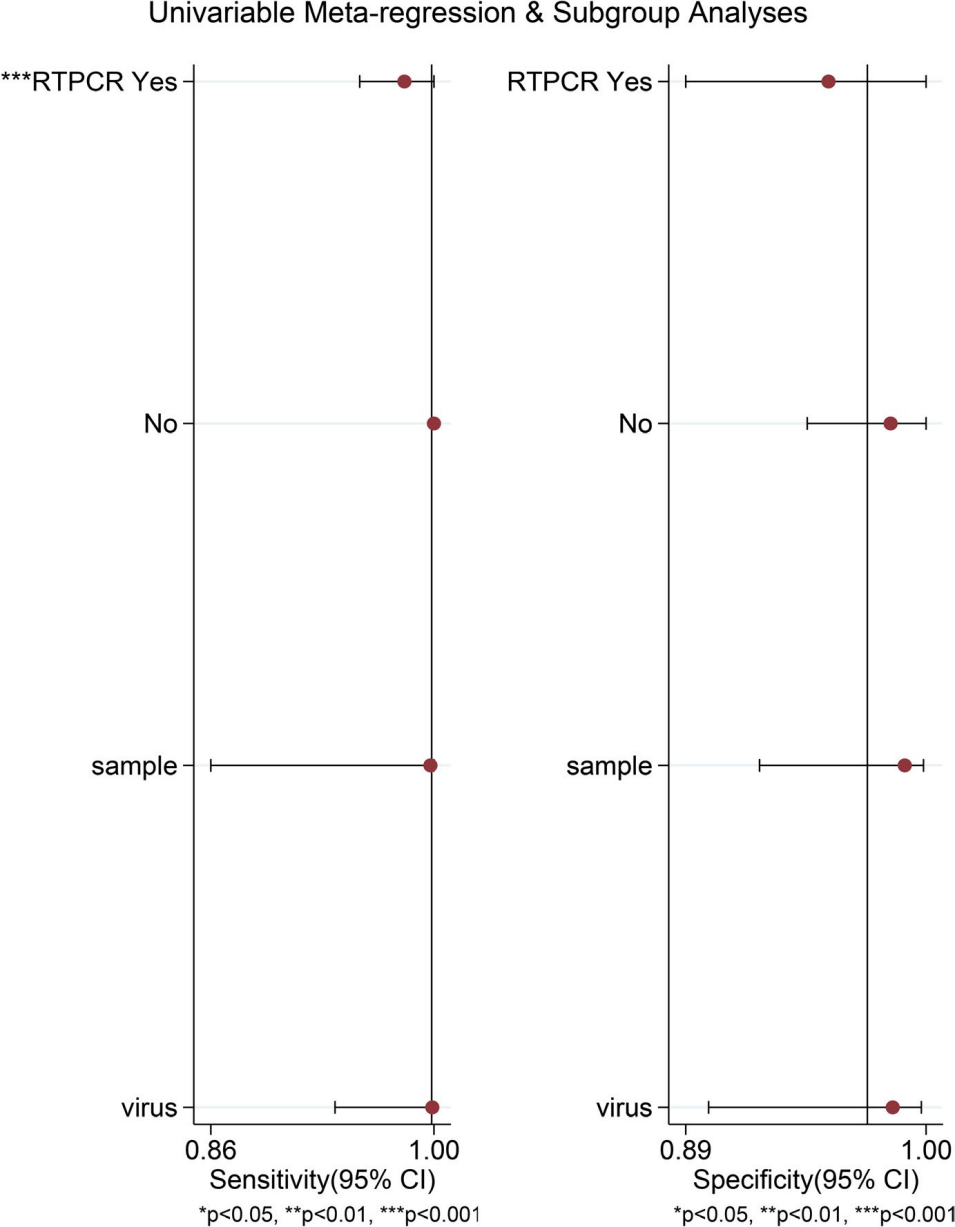
Meta‐regression results

It could be observed in Figure 5 that only the reference standard variable had statistical significance for sensitivity (*p* < 0.001). The results of the subgroup analysis were shown in Table 2.

**TABLE 2:** Subgroup analysis results

Variables	Subgroup	Number of studies	Sensitivity (95% CI)	*I* ^2^	Specificity (95% CI)	*I* ^2^
PCR technique	RT‐PCR	14	0.98 (0.92–1.00)	89.0%	0.98 (0.80–0.99)	94.1%
	Non‐RT‐PCR	4	1.00 (0.98–1.00)	0.0%	0.98 (0.92–1.00)	91.1%
Sample type	Fecal samples	5	1.00(0.81–1.00)	91.2%	0.82(0.37–0.97)	92.1%
	Pharyngeal swabs	3	0.99(0.81–1.00)	87.2%	0.97(0.71–1.00)	77.1%
	Fecal samples and Pharyngeal swabs	3	0.99(0.92–1.00)	4.5%	0.85(0.63–0.96)	87.5%

### 3.9 Publication bias

**FIGURE 6:**
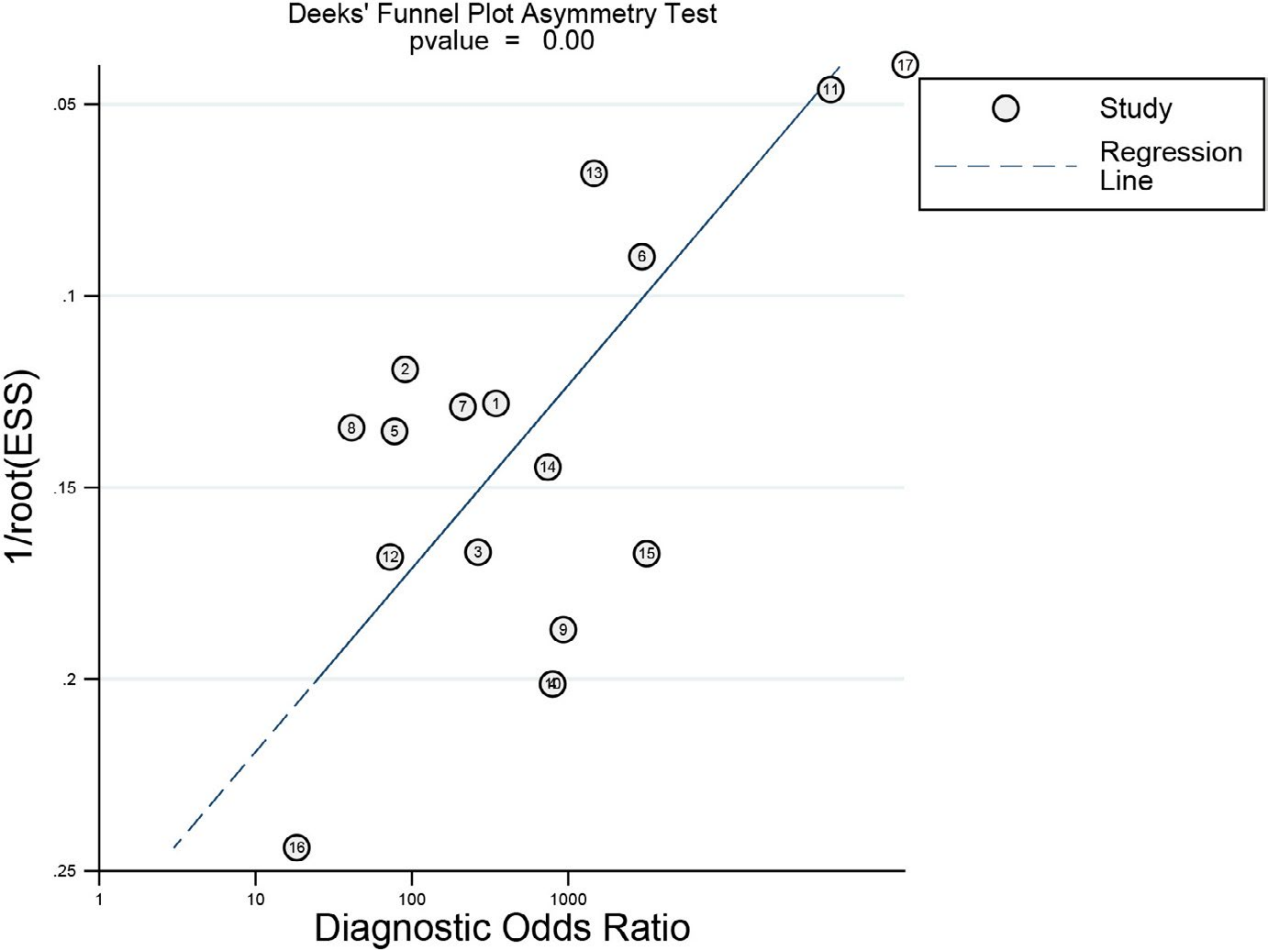
Deeks’ funnel plot asymmetry test to assess publication bias

As shown in Figure 6, significant publication bias was discovered in Deeks’ funnel plot (*p* = 0.00).

## 4 DISCUSSION

Hand, foot, and mouth disease poses a heavy disease burden mainly in Asia and the Pacific Rim, especially among children under the age of 5. In some severe cases, it can result in death due to fatal cardiopulmonary and neurological complications.^34^ Thus, it is of great significance to master rapid and effective diagnostic methods for the treatment of HFMD. Our study concentrated on investigating the diagnostic value of the LAMP assay for HFMD.

After merging the results of 18 articles, we gained the merged sensitivity, specificity, PLR, NLR, and DOR, which were 1.00, 0.97, 11.17, 0.05, and 538.12, respectively. DOR was so high that it was obvious that LAMP had a high diagnostic value for HFMD. Nevertheless, the following items also proved the point. First, the SROC curve was far away from the middle diagonal and lay close to the upper left corner. At the same time, its AUC was 1.0. Second, NLR < 0.1 and PLR > 10, suggesting that LAMP was reliable in judging whether a person had HFMD. Third, high sensitivity and specificity suggested that the possibility of misdiagnosis and missed diagnosis were both low. Nevertheless, it was found that higher sensitivity and specificity existed in the group that used both pharyngeal swabs and fecal samples than the one that used just one of them, which indicated that various samples might improve the diagnosis value. Additionally, compared with the group that took RT‐PCR as a reference standard, the non‐RT‐PCR group had higher sensitivity and specificity, which indicated that using non‐RT‐PCR as a reference standard might overstate the diagnosis accuracy.

Typical diagnostic methods for HFMD are virus isolation and serological detection. However, they are too time‐consuming to meet the requirement of treatment of patients in hospital. Recently, PCR‐based method is generally used for laboratory diagnosis of HFMD. However, it relies on operator skills and expensive instruments, which is difficult to be wisely used especially in developing countries.^35^, ^36^ Compared to the methods discussed above, LAMP has some outstanding features, such as a wide detection range and simpler equipment. The efficiency of LAMP is almost impervious to the presence of non‐target genomic DNA in the reaction, which is appropriate to the improvement of diagnostic systems.^10^, ^37^ Moreover, this meta‐analysis reveals that the sensitivity and specificity of LAMP for HFMD are high. According to this meta‐analysis, it can be inferred that LAMP has the potential to be an alternative diagnostic tool for HFMD in the laboratory. It can be applied early in various fields and contribute to control the spread of infection. Nevertheless, it can also be well popularized and equipped in economically underdeveloped areas, which is helpful for the rapid control of HFMD epidemic in these areas.^37^

Regarding heterogeneity, it was apparent that the heterogeneity caused by the effect threshold probably did not exist. However, the high heterogeneity derived from the non‐effect threshold could not be overlooked. In the random effects model, *I* ^2^ values of pooled DOR, sensitivity, specificity, PLR, and NLR were 56.2%, 94.0%, 95.9%, 88.9%, and 52.9% (*p* < 0.05), which exceeded 50%. Based on the results of meta‐regression, it could be observed that only the reference standard variable had statistical significance for sensitivity (*p* < 0.001), indicating that the sensitivity results obtained by studies using the gold standard of RT‐PCR and non‐RT‐PCR had statistically significant differences. Therefore, it was believed that the reference standard was a noticeable source of heterogeneity. Subsequently, a subgroup analysis was also performed. The heterogeneity of sensitivity in the non‐RT‐PCR subgroup decreased to 0, while another subgroup still exceeded 50% (decreased to 89.0%). It indicated that there were some extra potential sources of heterogeneity, such as the severity of the disease, different operators or conditions of the test, age or gender of the patients, and sample size.

Moreover, Deeks’ funnel plot was made to test publication bias. It showed that significant publication bias existed in our study (*p* < 0.05), which probably resulted from some unpublished negative results. That is to say, we were supposed to draw a conclusion based on the merged results with prudence.

Our study still had some limitations. First, the articles we included all originated in China, so the lack of data from other countries might impact the results. Second, some studies were considered to have a high risk of bias in certain domains, which might increase the risk of bias. Studies that did not refrain from a case‐control design might change the diagnostic accuracy.^38^ In Xia's and Zhao's study, they did not pre‐specify a threshold for the index test, which might result in measurement bias. In Xia's study, interpretation of reference standard results with knowledge of the index test results might also increase the measurement bias. Third, the sources of high heterogeneity were not figured out completely. Besides, it was unclear whether unpublished negative results affected the existing results significantly.

In conclusion, according to the present combined results, LAMP has high sensitivity and specificity for the diagnosis of HFMD. That is to say, it has a high diagnostic value for HFMD. With the characteristic of high efficiency and convenience, LAMP may become the main laboratory diagnostic tool for HFMD in the future. However, more high‐quality research is required to prove this conclusion.

## CONFLICT OF INTEREST

The authors declare that there are no competing interests associated with the manuscript.

## Supporting information

Fig S1

Fig S2

Fig S3

Fig S4

## Data Availability

Not applicable.
